# Comparison of glucose derivatives effects on cartilage degradation

**DOI:** 10.1186/1471-2474-11-162

**Published:** 2010-07-15

**Authors:** Thanyaluck Phitak, Peraphan Pothacharoen, Prachya Kongtawelert

**Affiliations:** 1 Thailand Excellence Center for Tissue Engineering and Stem Cells, Department of Biochemistry, Faculty of Medicine, Chiang Mai University, Chiang Mai, 50200, Thailand

## Abstract

**Background:**

Glucosamine (GlcN) is a well-recognized candidate for treatment of osteoarthritis. However, it is currently used in derivative forms, such as glucosamine-hydrochloride (GlcN-HCl) or glucosamine sulfate (GlcN-S). However, the molecular mode of action remains unclear. In this study, we compared the effects of Glucose (Glc), Glucuronic acid (GlcA), Glucosamine hydrochloride (GlcN-HCl) and Glucosamine sulfate (GlcN-S) on cartilage degradation.

**Methods:**

Porcine cartilage explants were co-cultured with recombinant human IL-1β and each tested substance for 3 days. HA, s-GAG and MMP-2 releases to media were measured using ELISA, dye-binding assay and gelatin zymography, respectively. Similar studies were performed in a human articular chondrocytes (HAC) monolayer culture, where cells were co-treated with IL-1β and each reagent for 24 hours. Subsequently, cells were harvested and gene expression measured using RT-PCR. All experiments were carried out in triplicate. Student's t-tests were used for statistical analysis.

**Results:**

In cartilage explants treated with IL-1β, GlcN-S had the highest chondroprotective activity of all four chemicals as shown by the inhibition of HA, s-GAG and MMP-2 released from cartilage. The anabolic (aggrecan core protein; AGG, SOX9) and catabolic (MMP-3, -13) genes in HACs treated with IL-1β and with/without chemicals were studied using RT-PCR. It was found that, GlcN-HCl and GlcN-S could reduce the expression of both MMP-3 and -13 genes. The IL-1β induced-MMP-13 gene expression was decreased maximally by GlcN-S, while the reduction of induced-MMP-3 gene expression was greatest with GlcN-HCl. Glc and GlcA reversed the effect of IL-1β on the expression of AGG and SOX9, but other substances had no effect.

**Conclusion:**

This study shows that glucosamine derivatives can alter anabolic and catabolic processes in HACs induced by IL-1β. GlcN-S and GluN-HCl decreased induced MMP-3 and -13 expressions, while Glc and GlcA increased reduced-AGG and SOX9 expression. The chondroprotective study using porcine cartilage explant showed that GlcN-S had the strongest effect.

## Background

Osteoarthritis (OA) is the most common form of arthritis, and is a public health problem throughout the world. OA is characterized by cartilage deterioration, as evidenced by quantitative and qualitative modification of proteoglycans (PGs) and collagen. An imbalance between the biosynthesis and the degradation of matrix components leads to a progressive destruction of the tissue, resulting in extensive articular damage [1].

Glucosamine (GlcN) is becoming increasingly popular as an alternative treatment for OA. GlcN is an aminosaccharide, acting as a preferred substrate for the biosynthesis of glycosaminoglycan chains and subsequently, for the production of aggrecan and other proteoglycans found in cartilage [2]. There is evidence that GlcN is equally effective or even better in decreasing pain in patients with knee OA, as compared to low dose Non-Steroidal Anti-Inflammatory Drug (NSAID) use [3,4]. Several clinical studies have indicated that crystalline GlcN-S is effective in controlling OA symptoms and disease progression [5-7]. In addition, the study of GlcN levels in plasma and synovial fluid suggests that GlcN is bioactive both systemically and at the site of action (joint) after oral administration of crystalline GlcN-S [8]. Although the treatment of OA with GlcN is quite popular, the exact mechanism of its effects on cartilage and chondrocytes, especially at the molecular level, remains unknown. There are many reports demonstrating the effect of GlcN and suggesting that GlcN reverses the decrease in proteoglycan synthesis and in UDP-glucuronosyl-transferase I mRNA expression induced by IL-1β [9]. Moreover, addition of GlcN to rat chondrocytes treated with IL-1β decreased the activation of the nuclear factor κB, but not the activator protein-1; GlcN can also increase the expression of mRNA encoding the type II IL-1β receptor (a decoy receptor) [10]. In human osteoarthritic chondrocytes, it was found that GlcN-S inhibits the synthesis of proinflammatory mediators stimulated by IL-1β through a NFκB-dependent mechanism [11]. Furthermore, the study of anabolic and catabolic gene expression in human osteoarthritic explants revealed that GlcN-HCl and GlcN-S downregulated both anabolic and catabolic gene expression [12]. Thus, the therapeutic effects of GlcN may be due to anti-catabolic activities, rather than due to anabolic activities.

GlcN used for OA treatment is mostly GlcN derivatives, such as GlcN-HCl and Glc-S. There are some reports that compare the effects of these derivatives. It was found that GlcN-S is a stronger inhibitor of gene expression than GlcN-HCl [13]. However, there has to date been no comparison of the chondroprotective effects of GlcN derivatives. In this study, we compared the chondroprotective effects of GlcN-HCl, GlcN-S, Glc and GlcA in porcine cartilage explants and human articular chondrocytes (HAC) that had been induced by IL-1β. Since the metabolic imbalance in OA includes both an increase in cartilage degradation and insufficient reparative or anabolic response [14], the effects of these glucose derivatives, on both catabolic and anabolic gene expression, were studied and compared in HAC treated with IL-1β.

## Methods

### Chemicals

The following chemicals were purchased from Sigma-Aldrich (USA): D-(+)-Glucose, D-(+)-Glucuronic acid γ-lactone, and D-(+)-Glucosamine hydrochloride. GlcN-S was obtained from Rottapharm and IL-1β was purchased from R&D (R&D system, USA).

### Preparation and treatment of cartilage explants

The cartilage degradation model was performed using porcine cartilage explant induced inflammation using IL-1β as described previously [15-17]. Briefly, the metacarpophalangeal joints were dissected for articular cartilage from 20-24 week-old pigs. These were then incubated in serum-free Dulbecco's modified Eagle's medium (DMEM) containing 200 units/ml of penicillin and 200 ug/ml of streptomycin with 5% CO_2 _and at 37°C. The explants were kept for 24 hours. Recombinant human interleukin-1β (25 ng/ml) was used to induce cartilage degradation. The explants were subsequently co-treated with IL-1β and various concentrations of Glc, GlcN-S, GlcA and GlcN-HCl (20, 40, 80 mM). The media were collected on the third day of the treatment and stored at -20°C for further analysis. It should be noted that all experiments were performed in triplicate using tissue from one animal donor.

### HAC culture and treatment

Primary chondrocytes were isolated from the non-inflammatory human cartilage that was collected after arthroscopic diagnosis of flat-pad syndrome patients at the Maharaj Nakorn Chiang Mai Hospital. Fully informed written consent was obtained from each patient and the study was approved by the Research Ethics Committee 3, Faculty of Medicine, Chiang Mai University (ethics approval code is 070CT111016). The cartilage was digested with trypsin at 4°C for 12 hours, and with collagenase (Sigma-Aldrich^®^, type IA) at 37°C for 3 hours. Then, the cells were washed with phosphate-buffered saline (PBS) and grown at high density in a monolayer culture comprising 10% fetal calf serum (FCS) DMEM. After the fourth cycle, the human chondrocytes were maintained in serum-free DMEM for 24 hours, prior to 24 hours of co-treatment with 10 ng/ml IL-1β and various concentrations (5, 10, 20 mM) of Glc, GlcN-S, GlcA and GlcN-HCl. Moreover, the expressions of interested genes (MMP-3, MMP-13, AGG and SOX9) were also investigated for the comparison between fresh primary isolated chondrocyte (P0) and its fourth passages.

### Cytotoxic study using the MTT assay

HAC (1 × 10^4 ^cells) were plated in triplicate in 96-well-plates and incubated overnight. Cells were treated with different concentrations (5, 10, 20 mM) of Glc, GlcN-S, GlcA and GlcN-HCl for 24 hours. After incubation, culture media were discarded, replaced with new culture media which contained 10% of 5 mg/ml MTT (3,[4,4-dimethy thiazol-2-yl]-2,5-diphenyl-tetrazolium bromide), discarded again, and followed by adding 0.2 ml of dimethyl sulfoxide (DMSO) to each well to solubilize the formed formazane crystals. The absorbance was measured at 540 nm using a microplate reader.

Percent of cell survival was calculated as follows:

### Measurement of s-GAG concentration

The concentrations of s-GAG in the conditional media were measured using dimethylmethylene blue (DMMB) [18] and a standard of shark cartilage chondroitin sulfate C (Sigma-Aldrich^®^, USA). The DMMB solution was used to dilute the sample, the standards and the appropriate blank solution. The absorbance of the resulting solution was measured at 525 nm using a microplate reader spectrophotometer. The levels of the ECM biomolecules released from the cartilage due to induction by IL-1β were calculated as:

%change={[(mediumfromD3)-(mediumfromD0)]/(mediumfromD0)}×100

where *D0 *and *D3 *are media collected on the start day and the third day, respectively.

### Detection of uronic acid

The concentrations of remaining glucuronic acid (GlcUA) in the explants were measured by a colorimetric assay using m-hydroxydiphenyl as a reagent [19]. Explants were digested by papain prior to measurement. The percentage of remaining uronic acid was calculated as:

### Measurement of HA concentration

HA concentrations were measured using the competitive inhibition-based enzyme-linked immunosorbent assay (ELISA) method using the commercialized AWHA Test kit [20] (Allswell Singapore Pte., Singapore) according to the manufacturer's instructions.

### Gelatin zymography

Pro-MMP-2 in the conditioned medium was detected by gelatin zymography as previously described [21]. The samples were subjected to sodium dodecyl sulfate polyacrylamide gel electrophoresis (SDS-PAGE) using 10% acrylamide gel containing 0.1 mg/ml of gelatin (Sigma-Aldrich^®^, USA) at 4°C under non-reducing conditions. After electrophoresis, SDS in the gel was removed by rinsing with 2.5% Triton-X 100 pH 7.5. The gel was then incubated at 37°C in the buffer (50 mM Tris-HCl, 5 mM CaCl_2_, 1 μM ZnCl_2_, 0.02% NaN_3_) for 18 hours and then stained with 0.1% Coomassie brilliant blue R250 (Bio-Rad Laboratories, Hercules, CA) in 50% methanol/10% acetic acid, and destained with 10% acetic acid/50% methanol. Finally, a Scion image densitometer was utilized to analyse the gelatinolytic activity.

### Gene expression analysis

RNA was extracted from the monolayer cells using an Aurum total RNA purification kit (Bio-Rad Laboratories, Hercules, CA, USA). Using the RevertAid™ First Stand cDNA synthesis kit (MBI Fermentas, Germany), the net sum RNA (500 ng) of each sample was reverse transcribed into complementary DNA (cDNA). Primer and probe sets were designed using Primer Express 2.0 software (Applied Biosystems, Foster City, CA, USA) and nucleotide sequences are: AGG; 5' ACTTCCGCTGGTCAGATGGA3' 3' CAACACTGCCAACGTCCAGAT5', SOX9; 5' ACACACAGCTCACTCGACCTTG 3', 3' GGAATTCTGGTTGGTCCTCTCTT 5', MMP-3; 5' TTTTGGCCATCTCTTCCTTCA 3', 3' TGTGGATGCCTCTTGGG TATC5', MMP-13; 5' TCCTCTTCTTGAGCTGGACTCATT 3', 3' CGCTCTG CAAACTGGAGGTC 5', GAPDH; 5' GAAGGTGAAGGTCGGAGTC3', 3' GAAGATGGTGATGGGATTTC 5'. The amplified products were separated by electrophoresis on 2% (w/v) agarose gels, stained with ethidium bromide and then imaged using a Bio-Rad Gel-Doc fluorescent image analyzer. To allow semi-quantitative comparisons of mRNA levels, the integrated densities were calculated by the Scion Image analysis software and divided by levels of the house-keeping gene GAPDH (glyceraldehydes-3-phosphate dehydrogenase) as previously described [22,23].

### Statistical analysis

All data were shown as mean ± SD and the statistical analysis was performed using t-tests. P-values less than 0.05 were considered significant.

## Results

### Chondroprotective effects of Glc, GlcN-S, GlcA and GlcN-HCl in porcine cartilage explants

Porcine cartilage explants were induced to degrade by using 25 ng/ml IL-1β and the chondroprotective effects of all four chemicals were studied by co-treating the explants with IL-1β and each chemical (20, 40, 80 mM) for 3 days. Conditioned media were collected and analyzed. Many molecules were used to be the indicators. Interleukin-1 beta induces the degradation of extracellular matrix (ECM) molecules in cartilage discs, and degraded ECM molecules will be released into the media while undegraded molecules will remain in the cartilage tissue.

The release of HA and s-GAG, which are ECM molecules, from cartilage tissue into media were analyzed by ELISA and dye-binding assays, respectively. Gelatin zymography was used to measure MMP-2 activity. The remnant ECM molecules were measured by digestion of conditioned cartilage with papain, followed by measuring the remaining uronic acid.

**Figure 1:**
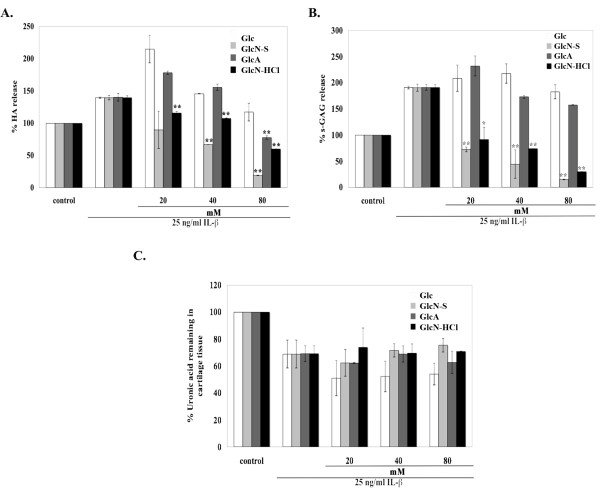
**The effects of Glc, GlcN-S, GlcA and GlcN-HCl: release of s-GAG, HA from porcine cartilage tissues to the media, the uronic acid remaining in the cartilage tissue**. Porcine cartilage explants were cultured with IL-1β (25 ng/ml) in absence and presence of each chemical (at varying concentrations of 20, 40, 80 mM) for 3 days. In the media, the s-GAG release was measured by using a dye-binding assay, and HA release was measured by ELISA. Cartilage discs were digested with papain and then the uronic acid content was measured. *, ** Denotes: a value that is significantly different (p < 0.05 and p < 0.01, respectively) from the IL-1β control.

**Figure 2:**
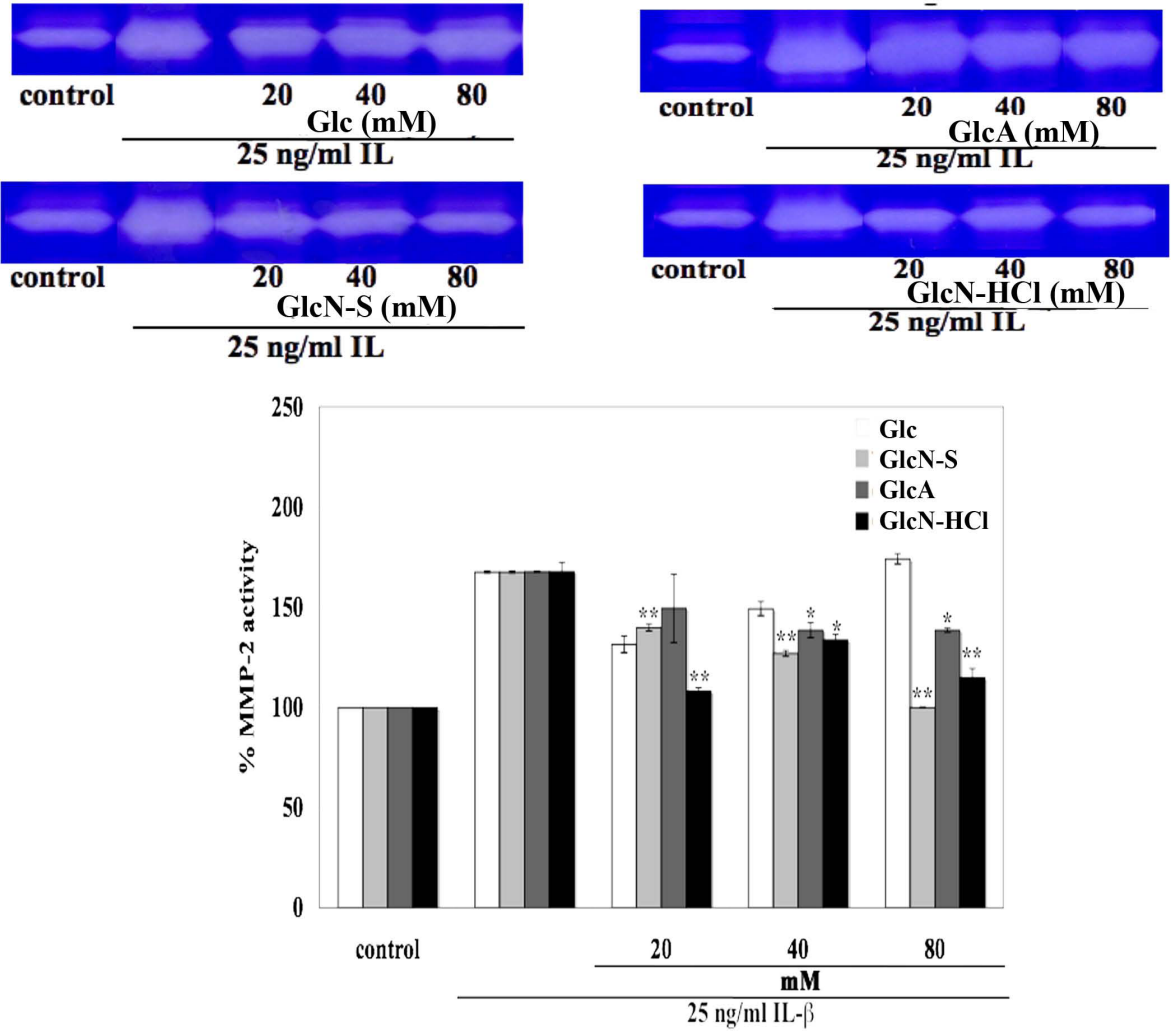
**Effects of Glc, GlcN-S, GlcA and GlcN-HCl on the production of MMP-2**. Porcine cartilage explants were cultured with IL-1β (25 ng/ml) in the absence and presence of each chemical (at varying concentrations of 20, 40, 80 mM) for 3 days. Media were collected and were then analyzed by gelatin zymography as described in the text. Three experiments were carried out independently and they were reproducible. *, ** Denotes a value that is significantly different (p < 0.05 and p < 0.01, respectively) from the IL-1β control.

IL-1β induced the release of HA and s-GAG from cartilage into media (Figure 1A, B). GlcN-S, GlcN-HCl and GlcA decreased HA release. Among these three chemicals, GlcN-S exhibited the highest inhibitory effect. However, HA released to the media was not reduced by Glc (Figure 1A). For s-GAG releases, GlcN-S and GlcN-HCl had the ability to reduce s-GAG release while Glc and GlcA did not. GlcN-S also had the highest effect on s-GAG. Moreover, IL-1β induced the activity of MMP-2 (Figure 2). This induced activity was decreased by GlcA, GlcN-HCl and GlcN-S, but not by Glc. Similarly to HA and s-GAG, GlcN-S possessed the highest inhibitory effect.

For the remaining uronic acid in cartilage discs, when the cartilage discs were treated with IL-1β, the remaining uronic acid content was lower than that of the control group (Figure 1C). All four chemicals did not significantly reverse this effect of IL-1β, but GlcN-S and GlcN-HCl tended to inhibit uronic acid loss from cartilage at the highest dosage (80 mM). Altogether, these results suggest that GlcN-S had the highest chondroprotective effect among all four chemicals used in our porcine cartilage explant model. We continued by analyzing the chondroprotective effects of all four chemicals in human chondrocytes.

### Cytotoxic effects of Glc, GlcN-S, GlcA and GlcN-HCl in HAC

**Figure 3:**
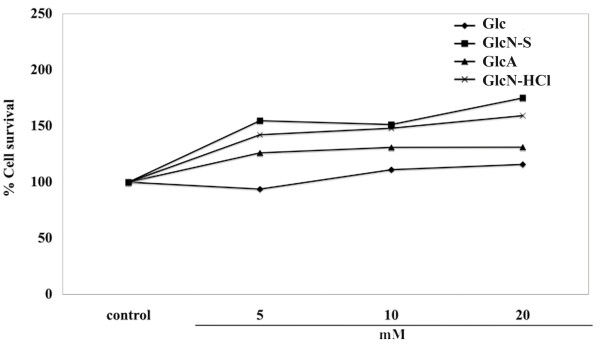
**The cytotoxic effects of Glc, GlcN-S, GlcA and GlcN-HCl**. The cytotoxic effect of all reagents at concentrations 5, 10 and 20 mM in human articular chondrocytes were studied by the MTT assay.

The cytotoxic effects of 5, 10 and 20 mM of all four chemicals were initially studied in the HAC model. As shown in Figure 3, none of the three concentrations of the four chemicals had cytotoxic effects on HAC. Thus, these three concentrations could be used for studying the chondroprotective effects of the chemicals in subsequent experiments.

### Effects of Glc, GlcN-S, GlcA and GlcN-HCl on HA release and MMP-2 activity in IL-1β-treated-HAC

HACs were co-treated with 10 ng/ml IL-1β and 5, 10 and 20 mM of each chemical for 24 hours. The conditioned media were collected and analyzed for HA and MMP-2 activity.

**Figure 4:**
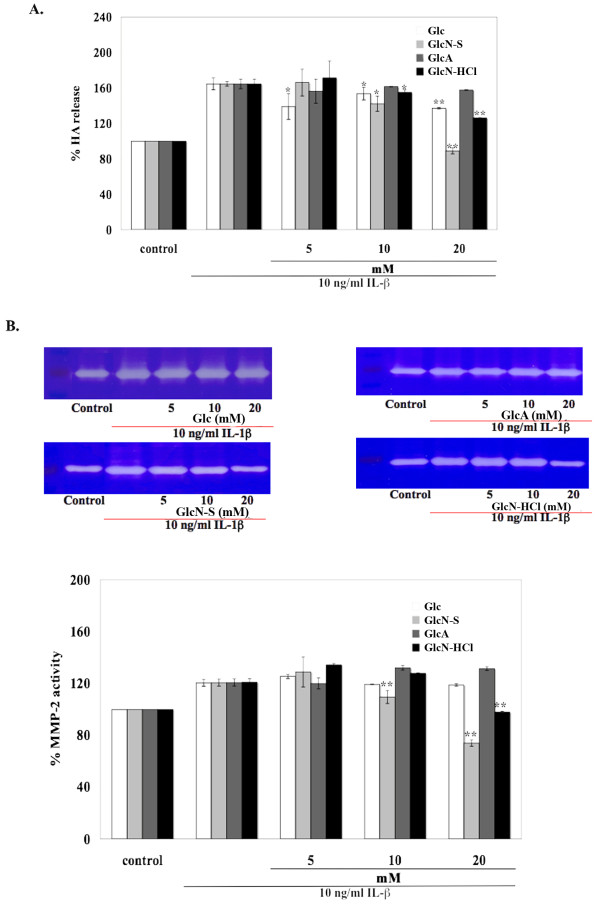
**Effects of Glc, GlcN-S, GlcA and GlcN-HCl on the release of HA (A), s-GAG (B) and MMP-2 (C) from chondrocytes**. Chondrocytes were co-treated with 10 ng/ml IL-1β and various concentrations of each chemical (5, 10, 20 mM) for 24 hours. The conditioned media were analyzed for HA, s-GAG and MMP-2 activity as described in the Experimental section. *,** Denotes a value that is significantly different (p<0.05 and p<0.01, respectively) from the IL-1β control.

IL-1β was able to induce the release of HA and MMP-2 activity into the media (Figure 4). The induced released HA was inhibited by GlcN-S, Glc and GlcN-HCl but was not inhibited by GlcA. GlcN-S showed the highest inhibitory effect, followed by Glc and GlcN-HCl (Figure 4A). Glc and GlcA did not decrease induced MMP-2 activity, whereas both GlcN-S and GlcN-HCl did so. Among the four chemicals studied, GlcN-S had the highest inhibitory effect (Figure 4B). In the human chondrocyte model, GlcN-S also had the highest chondroprotective effect.

### Effects of Glc, GlcN-S, GlcA and GlcN-HCl on catabolic gene expression in HAC

To study gene expression, HACs were co-treated with 10 ng/ml IL-1β and 5, 10 and 20 mM of each chemical for 24 hours. Cells were harvested and then extracted for mRNA, which was used to synthesize cDNA. The RT-PCR was performed using the primers as described in Methods.

**Figure 5:**
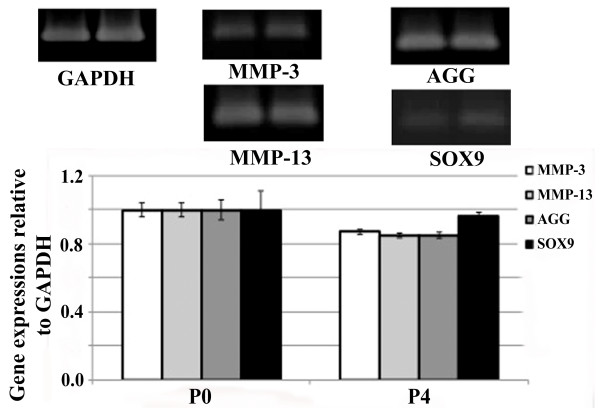
**The mRNA expression of MMP-3, MMP-13, AGG and SOX9 in fresh isolated chondrocytes (P0) and in the fourth cultured passage chondrocytes (P4)**. Passage 0 and P4 confluent human chondrocytes in 25-cm^3 ^flasks were cultured in serum free-DMEM for 24 hours. Cells were harvested and gene expression was analyzed. MMP, matrix metalloproteinase, AGG, aggrecan; SOX9, SRY-type HMG box.

It has been well documented that the expression levels of many genes change in cultured chondrocytes as compared to that in intact cartilage and, moreover, between the cultured passages [24-27]. To avoid the variations of the expression levels between passages, the genes showing negligible change between passage cultures were chosen for further investigation. There was a report found that the expression of MMP-3, MMP-13, aggrecan core protein (AGG) and SOX-9 (a transcriptional factor for type II collagen) were not significantly changed between fresh isolated chondrocytes (passage 2) and used passage (passage 4) [28]. In agreement with previous studies, we found that MMP-3, MMP-13, AGG and SOX9 mRNA expressions in P4 cultured chondrocytes were not significantly different with fresh isolated chondrocytes (Figure 5). Due to there was no variation of these genes between P0 and P4 cultured chondrocytes, thus MMP-3, MMP-13, AGG and SOX9 gene expressions were chosen for further investigation.

**Figure 6:**
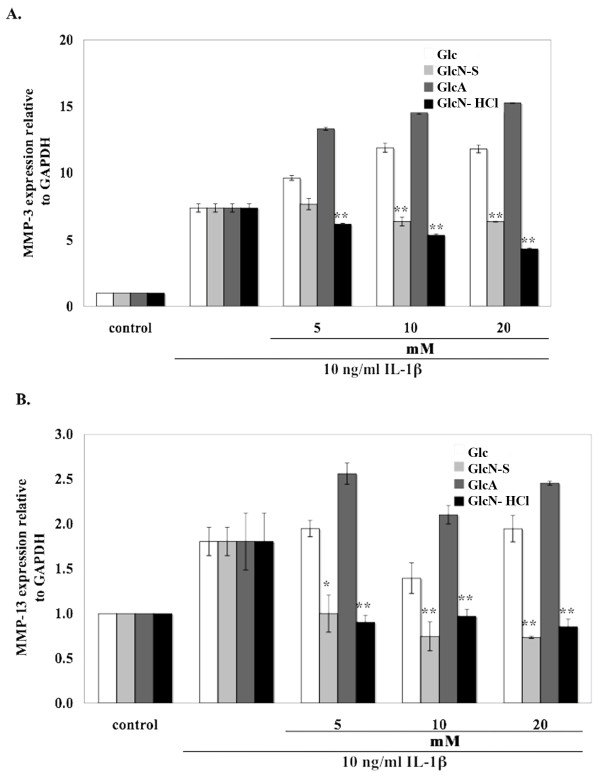
**Effect of Glc, GlcN-S, GlcA and GlcN-HCl on the mRNA expression of proteinases [MMP-3 (A), -13 (B)]**. Confluent human chondrocytes in 25-cm^3 ^flasks were cultured with IL-1β (10 ng/ml) in the presence and absence of each chemical for 24 hours. Cells were harvested and gene expression was analyzed. MMP, matrix metalloproteinase. *, ** Denotes a value that is significantly different (p < 0.05 and p < 0.01, respectively) from the IL-1β control.

Regarding the expression of catabolic genes, we studied MMP-3 and MMP-13. Both MMP-3 and -13 gene expressions were induced by IL-1β (Figure 6). The induced MMP-3 expression was inhibited by GlcN-HCl and GlcN-S, while GlcN-HCl showing the highest inhibitory effect. Glc and GlcA had no inhibitory effect on expression of either gene. On the contrary, Glc and GlcA seemed to further induce MMP-3 expression. For MMP-13, GlcN-HCl and GlcN-S could inhibit the induction of MMP-13 by IL-1β, and GlcN-S showed the highest inhibitory effect. Glc had no effect on induced MMP-13 expression, but GlcA increased MMP-13 expression.

### Effects of Glc, GlcN-S, GlcA and GlcN-HCl on anabolic gene expressions in HAC

**Figure 7:**
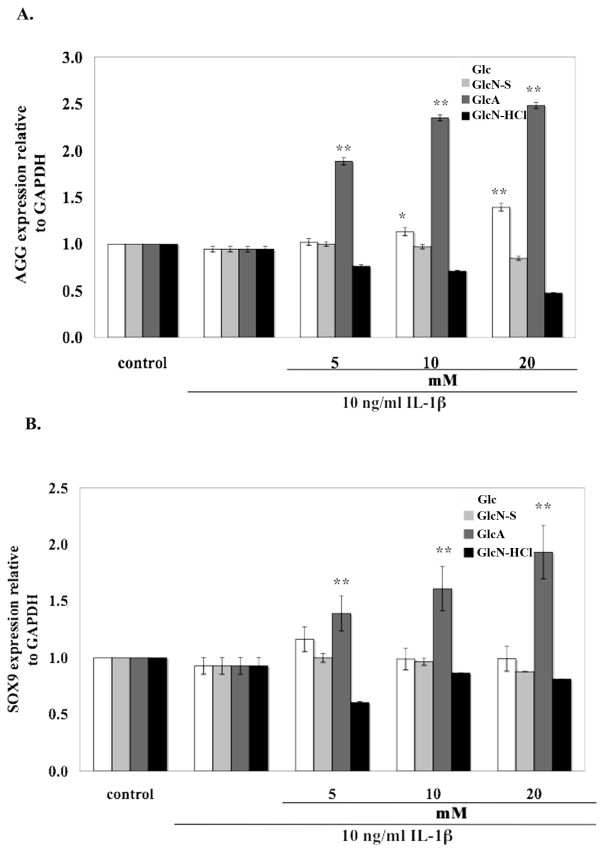
**Effects of Glc, GlcN-S, GlcA and GlcN-HCl on the mRNA expression of cartilage genes [AGG (A), SOX9 (B)]**. Confluent human chondrocytes in 25-cm^3 ^flasks were cultured with IL-1β (10 ng/ml) in the presence and absence of each chemical for 24 hours. Cells were harvested and gene expression was analyzed. AGG, aggrecan; SOX9, SRY-type HMG box. *, ** Denotes a value that is significantly different (p < 0.05 and p < 0.01, respectively) from the IL-1β control.

Regarding the effects of the tested compounds on anabolic genes, we analyzed the expression of AGG and SOX9. There was no significant difference in expression of either AGG or SOX9 genes when treated with IL-1β (Figure 7). Glucose and GlcA induced AGG gene expression, while GlcN-HCl and GlcN-S showed reduced effects. The GlcN-HCl group had the highest effect. SOX9 expression was not changed when treated with Glc or GlcN-S, but was increased by GlcA and was decreased by GlcN-HCl.

## Discussion

Osteoarthritis (OA) is the most common form of arthritis, affecting millions of people worldwide [29]. It remains the major cause of disability in the elderly, affecting about 60% of men and 70% of women above the age of 65. As regards therapeutic strategies for OA, there are a large number of active research and drug discovery programs aimed to identify structure-modifying ways to inhibit joint destruction in OA, and existing drug therapies to reduce symptoms. None of these approaches, however, has significant efficacy as a disease modifying anti-OA drug [30]. Until recently, COX-2 inhibitors were widely used to provide symptomatic relief, but the increased risk of heart attacks and strokes associated with these drugs led to the recall of some products from the market and warnings concerning their use [31,32]. As alternatives to non-steroidal anti-inflammatory drugs (NSAIDs) and COX-2 inhibitors, other symptom-modifying drugs currently in clinical trials for OA include nitric oxide-releasing analgesics, bradykinin B2 receptor antagonists and capsaisin analogues [33]. Other treatments for OA could include intra-articular injection with long-acting corticosteroids or hyaluronan, which would also provide symptomatic relief. One recent alternative therapy is nutraceutical treatment. Glucosamine (GlcN) is becoming increasingly popular as an alternative treatment for OA. There is evidence that GlcN is equally effective or even better in decreasing pain in patients with knee OA, as compare to low dose NSAID use [3,4]. Furthermore, there are several reports showing that there was less joint space narrowing in people with knee OA who took GlcN compared to placebo, over a period of 3 years [34,35]. This suggests that that GlcN can delay the progression of knee OA. In the last several decades, there has been an increasing number of patients who have started using GlcN, with or without direction from a physician. Although the effectiveness of GlcN has been debated in a recent article, there are clinical studies suggesting that GlcN probably has structure modifying effects in patients with knee OA [34,35]. The underlying effects of GlcN on cartilage that are responsible for these clinical outcomes are still unclear. It has been proposed that addition of GlcN to chondrocyte cell cultures leads to more GAG production, since GlcN is the basic building block of GAG molecules. Some studies have supported this hypothesis [36-40]. However, there are studies that observed negative effects on GAG production after GlcN addition [10,41-45]. Apart from influencing matrix synthesis, several studies have shown that GlcN is also able to interfere with enzymatic matrix degradation [9,46-49]. These conflicting results can have various causes.

It is unclear whether or not GlcN-S, Glc-HCl and *N*-acetyl-glucosamine (GlcN-Ac) have similar effects on cartilage. Differences in the results can also be explained by the varieties of culture models used (e.g., monolayer or pellet culture) and the variations in culture duration.

No previous studies have examined the metabolism of GlcN in humans. However, there is one published investigation of synovial and plasma GlcN concentrations in OA patients following oral administration of crystalline GlcN-S. That study reported that GlcN is bioavailable both systemically and at the site of action (the joint) [8]. However, it is still unclear whether the administrated GlcN will be metabolized to Glc in humans, and whether metabolized Glc will have effects similar to those of GlcN. Thus, in this experiment, we studied and compared the effects of Glc, GlcA, GlcN-S and GlcN-HCl in two models using IL-1β to induce inflammtion: first in a porcine cartilage explant model, and second, in a human articular chondrocyte (HAC) culture model. In the porcine cartilage explant model, GlcN-S showed the highest chondroprotective effects (inhibited IL-1β's effect on HA and s-GAG degradation) followed by GlcN-HCl, while Glc and GlcA did not show these effects. In the HAC model, GlcN-S had the highest effect, shown by inhibition of IL-1β induced HA release and MMP-2 activity, followed by GclN-HCl and GlcA, but Glc had no effect. Thus in administration of glucose derivatives, if these reagents were metabolized to Glc, the metabolized compound might not have a chondroprotective effect.

There are also reports showing that GlcN decreases expression of both anabolic and catabolic genes in human OA cartilage explants [12]. We expected that GlcN-S would show the highest inhibitory effect on IL-1β, because it had the highest effect in porcine cartilage explants. But our results showed that only MMP-13 gene expression could be reduced by GlcN-S. For the other catabolic gene, MMP-3 was mostly inhibited by GlcN-HCl, inversely with Glc, and GlcA further induced both MMP-3 and -13 expression in HAC treated with IL-1β. In anabolic gene expression, both AGG and SOX9 gene expression were not significantly changed by IL-1β. AGG expression was induced by Glc and GlcA, but was reduced by GlcN-S and GlcN-HCl. SOX9 expression was increased by GlcA and decreased by GlcN-HCl. Altogether, it seemed that Glc and GlcA could induce both catabolic and anabolic gene expression while GlcN-S and GlcN-HCl reduced the expression of the catabolic genes.

## Conclusion

Our results illustrate two key points. Firstly, from the chondroprotective study, GlcN-S had the most significant effect. However, at the mechanistic level of gene expression, GlcN-S had the strongest effect only on MMP-13 expression. Secondly, GlcN-HCl and GlcN-S showed significant effects on catabolic gene expression but not on anabolic genes, whereas Glc and GlcA had significant effects on anabolic genes but not on catabolic genes. However, if we consider chondroprotective effects, GlcN-HCl and GlcN-S were more effective than Glc and GlcA. Here, we used normal HAC and induced inflammation by IL-1β to mimic the onset of OA. These results thus demonstrate the importance of inhibition of catabolic genes in the onset of the disease.

## Abbreviations

OA: Osteoarthritis; Glc: Glucose; GlcN: Glucosamine; GlcA: Glucuronic acid; GlcN-HCl: Glucosamine hydrochloride; GlcN-S: Glucosamine sulfate; HA: hyaluronic acid; s-GAG: sulfated glycosaminoglycan; HAC: human articular cartilage; MMP: matrix metalloproteinase

## Competing interests

The authors declare that they have no competing interests.

## Authors' contributions

TP performed most of the experiments, data analysis and manuscript preparation. PP performed some experiments and participated in the study design and data analysis. PK conceived and developed the study design and participated in manuscript preparation. All authors read and approved the final manuscript.

## Pre-publication history

The pre-publication history for this paper can be accessed here:

http://www.biomedcentral.com/1471-2474/11/162/prepub
